# Observations on the Use of the Tinct. Ferri. Mur. in Scarlatina

**Published:** 1854-05

**Authors:** H. L. Byrd

**Affiliations:** Proffessor of Mat. Med. and Therapeutics, Savannah Medical College


From the Charleston (S. C.) Medical Journal and Review.

### *Observations on the use of the Tinct. Ferri. Mur. in Scarlatina.* By H. L. Byrd, M. D., Proffessor of Mat. Med. and Therapeutics, Savannah Medical College.

During the present year, both erysipelas and scarlatina have prevailed to some extent in this city, and the latter disease has been attended with considerable mortality among young children. After having used the tincture chloride of iron, in erysipelas, with considerable success, as recommended by Dr. Bell, (page 126 of your Journal for last year,) and remembering the general sequela of scarlatina—often after the most favorable convalescence—I determined to give it a trial in the latter disease My first impressions, as to its usefulness, were strengthened by the recolection, that chlorine had been recommended in scarlatina, and a knowledge of the value of mur. tinct. ferri, in the anasarcous swelling, which so often result after an attack of that disease. My most sanguine hopes have been more than realized, experimentally in more than twenty cases. So much am I convinced of its value, that I would not willingly exchange it for all the other remedies which I have heretofore used, or seen recommended, in scarlet fever. I am not aware of the article having been noticed before. Should such have been the case, however, I feel it due to the profession, and to suffering humanity, that the fact should be as widely disseminated as practicable. I will leave others to determine its *modus operandi* and proceed at once, to the narration of some of the most prominent cases, and the peculiar circumstances connected with them. The first case in which I used the mur. tinct. iron, occurred on the 10th July, 1853 The patient, a bright mulatto boy, aet. eight years, exhibited the eruption very dist nctly, over the entire surface of the body and extremities; the throat was very much injected, and the ulceration of the tonsils had already taken place. I ordered the bowels opened with salts, senna and manna, and the tincture of iron given afterwards, in eight drop doses diluted with gum water, every four hours. Gargle—1 drachm of the tincture to 4 ℥ of water, every three or four hours. This case went on well, and in two or three days the patient was up about the chamber. The second case was the son of H. C., Esq , aged two years. The child was rather delicate, with fair hair and full blue eyes. On my first visit, (20th July,) I found the skin covered with the eruption peculiar to scarlatina. The whole surface seemed to be involved in the eruption; the tonsils were red and injected: the parotids enlarged and painful to the touch, the left particularly so; the pulse 150, compressible; tongue furred, except at the edges, which were red; considerable thirst; no action of the bowels since the previous day. I directed a dose composed of ol ricin. ℥ iii. sup. carb. soda, grs. ii. tinct. opii camph. gutt. iv. camphor liniment to be rubbed over the enlarged glands of the neck, and a flannel saturated with the same, applied afterwards; throat penciled three times a day, with a solution of six grs. of argent nitrat. to one ounce rose water. After the action of the oil mixture, the following was directed to be given in teaspoonful doses, every two hours, until visited again, viz: ℞. Salt tart. ℈ i, mucil. G. acac, ℥ iv tart. ant. et Pot. gr. ⅛, tinct. op. camph. ℈ i, M; iced gum water allowed as a common drink. On my visit in the afternoon, I learned that the bowels had been acted upon once or twice, and the pulse was reduced by the last mixture, to 140, and the skin was soft, though not actually moist.

The child being delicate, and of somewhat strumous habit; and having been pleased with the success of the iron in the preceding case, and believing the indications even stronger in this one, I resolved to venture on its use at once. I directed mur. tinct. ferri. 50 drops, mucil g. arabic, ℥ ii, M. a teaspoonful to be given every four hours Suspend previous mixture and continue iced gum water.

On my visit the following morning, I found the skin less red; pulse 130; no action of the bowels since yesterday; urine somewhat increased in quantity; tongue clean and red; throat the same; glands the same; continue treatment to throat and neck; also, the iron as before. Evening visit—found the patient in much the same condition as at last visit: pulse 128. The next morning the skin less red; appetite increased; tongue clean and red; some thirst, which, however, is rapidly allayed by the iced gum water; neck and throat the same. Ordered the castor oil mixture mentioned above; directed the iron treatment to be resumed after its action. Did not call again until the following morning, at which visit I found that the bowels had acted well the previous day; condition generally improved: pulse 118. The iron was continued, and the lunar cau tic twice per day to the throat. The next morning the skin was much paler, the throat much improved, the pulse 110. The bowels being bound, a dose of castor oil was prescribed, and, after its action, the iron was resumed. The following morning, I found all the symptoms so much improved, that I did not deem it necessary to continue my visits.

The epidermis had commenced to desquamate, and the throat appeared well, beyond a slight redness, which remained for a few days, as I subsequently learned. The patient continued improving for near two weeks, when he was taken with slight fever, after one or two rides during the afternoon. During this attack, the glands of the neck, which had not entirely subsided from the first illness, became swollen and very painful, and despite of every effort, supperation took place on the left side. I opened the abscess, kept it poulticed with flaxseed, and put my patient on the syrup of the iodide of iron in six drop doses, four times per day, with one-half a grain sulphate quinine, three times per day, and in a few days he was well. I have been thus minute in the details of this case as it was the only one out of twenty in which suppuration occurred; and, further to convey a correct general idea of the course usually pursued. In no case did anasarca supervene, and the cases were usually cured in from seven to ten days. It is proper that I should remark, that occasionally other remedies, as blue pill, rhubarb, etc., were found to be necessary; and in all cases, with one or two exceptions, I made an effort to lessen the frequency of the pulse, and to determine to the surface before commencing with iron. Minute doses of tart. ant. or ipecac, in combination with other diaphoretics, were used for this purpose; and in all cases in which the tongue grew dry under the use of the iron, it was suspended until that difficulty was removed. I found but few cases, however, in which the dryness of the tongue was traceable to the iron. I used demulcents and cool drinks, as asked for, throughout the disease. In one case treated recently, the infant child of the Hon. E. J. H., I used no other remedy than the mur. tinct. of iron. It was given in doses of from one-half to three fourths of a drop suspended in gum arabic water three times per day. Notwithstanding the throat was considerably inflamed, and the glands enlarged, this patient convalesced rapidly, and was well in four or five days. The child was an infant at the breast, six or seven weeks old. Two other cases had previously occurred in the same family, in one of which I scarified the tonsils, and blistered the throat; finding both these expedients necessary to relieve the unusual inflammation and tumefaction of the tonsils which existed. It yielded readily to the iron, after the reduction of the high arterial excitement that ushered in the attack. In this case, complete desquamation of the epidermis occurred. The last case worthy of notice, is that of Ella, aged eight years, the eldest child of Dr. R. A., now under treatment (Dec. 2d). I saw her first, five days ago. The eruption was very distinct, and the throat considerably ulcerated; the tongue was coated with a brown fur, except the edges, which were red; the bowels constipated; pulse 145. I prescribed three grains each of mass hydrarg., pulv. rhei., and creta preparat. to be followed by castor oil in four hours. After the action of the oil on the bowels, the pulse remaining the same, and the skin dry, I used a mixture composed of sal. tartar, ʒ ss, mucil. g. acac. ℥ vi., tart. ant. et pot. gr. ⅛ and paregoric ʒ ss. in teaspoonful doses, every two hours, for ten or twelve hours; after which, the mur. tinct. iron was begun with and continued in doses of ten drops, three times per day, until this morning, when it was deemed no longer necessary. This case is alluded to, simply on account of the obstinate character of the ulceration and inflammation of the throat. I have used the mixture of silver in different proportions to the ounce of water without much benefit until yesterday, when a solution of twenty grains to the ounce was used. Gargles of tannin and pyroligneous acid, were also resorted to as auxiliaries during the intervals that the caustic was suspended.—A circumstance worthy of note was, that little or no swelling or enlargement of the glands of the neck occurred during the entire case. After the solution was used. as just stated, two or three times, without any perceptible improvement in the ulceration, I applied the solid nitrate of silver with complete success.

